# Molecular Research on Genes Involved in Metabolic Diseases

**DOI:** 10.3390/biomedicines11061671

**Published:** 2023-06-09

**Authors:** Francisco Lara-Hernandez, Luis Alvarez, Javier Chaves, Ana-Barbara Garcia-Garcia

**Affiliations:** 1 Genomic and Diabetes Unit, INCLIVA Biomedical Research Institute, 46021 Valencia, Spaina.barbara.garcia@ext.uv.es (A.-B.G.-G.); 2 Spanish Biomedical Research Centre in Diabetes and Associated Metabolic Disorders (CIBERDEM), ISCIII, 28029 Madrid, Spain

Numerous genes involved in different metabolic diseases have been identified, and this number is increasing. In addition, an increasing number of people are affected by various metabolic diseases, such as the different types of diabetes mellitus, insulin resistance, dyslipidaemias, etc., and other diseases not considered to be cardiometabolic. The identification of new genes and mechanisms is achieved using different strategies.

This Special Issue concerns the identification of and research on the mechanisms or genes involved in metabolic diseases, for which four articles have been published. These works cover different issues regarding metabolic diseases and strategies for the identification of mechanisms, including GWAS, the use of candidate genes, and epigenetic and functional studies. In the first paper, written by Bompada et al. [1], the authors used an epigenomic strategy based on the genome-wide enrichment of H3K9ac (Acetylated Histone 3 Lysine 9) via immunoprecipitation sequencing (ChIP-seq) in PBMCs to identify activated pathways and interactions present in patients with Type 2 Diabetes (T2D) and atherosclerotic diseases. The results showed important changes in regions with, and the levels of, H2K9ac in patients (about 80 gains and 405 losses), for which the enrichment in regions previously associated with T2D and T1D was remarkable. Some of the pathways were activated, and within these pathways, the activation of the *TCF7L2* and *HLA* genes was significant. An important aspect of the identified regions was their relation to immune response and its role in atherosclerosis development. This work revealed possible interactions between genetic and epigenetic mechanisms. 

The second work, written by Ferrández-Carrion et al. [2], identified a slight but statistically significant preference for sweetness among T2D patients. These data can be referenced with previous works showing a higher predilection for sweetness among diabetics [3]. Based on this preference and the lack of knowledge regarding the genetics of taste preferences [4], the cited group performed a GWAS. The results allowed them to identify several SNPs in certain genes, among which a remarkable SNP was identified, namely, the rs2091718 SNP located in the *PTPRN2* (Protein Tyrosine Phosphatase Receptor Type N2) gene, whose minor allele was associated with a reduced preference for sweetness [2]. *PTPRN2* is expressed in several tissues, including the brain (in the cerebral cortex, hippocampus, and pituitary gland), gut (in the stomach, duodenum, small intestine, colon, and rectum), pancreas, and kidneys (https://www.proteinatlas.org/ENSG00000155093-PTPRN2/tissue, accessed on 30 May 2023). *PTPRN2* would be relevant to T2D due to its role as a pancreatic islet autoantigen and a beta-cell function regulator, and therefore in insulin secretion via glucose [5,6,7]. The expression of this gene in the brain and digestive tract may be related to sugar preference and may evidence an association between *PTPRN2* and the gut–brain axis [8]. These data argue in favour of considering taste preference genetics as a factor related to T2D and the importance of the functional gut–brain axis in the appetitive effects of sugar. 

The third work, written by García-García et al. [9], demonstrated the importance of *SREBF2* in relation to glucose and cholesterol levels in the blood and their metabolism. The authors identified a very rare variant in the promoter of *SREBF2* in a large family via candidate gene approximation. After excluding mutations in the main genes related to Autosomal Dominant Hypercholesterolemia (ADH; namely, the *LDLR*, *APOB*, and *PCSK9* genes) and in the main genes related to Maturity Onset Diabetes of the Young (MODY; namely, *GCK*, *HNF1A*, and *HNF4A*), the authors analysed different genes related to cholesterol metabolism involved in the SREBP system (*SREBF2*, *SREBF1*, *MBTPS1*, *MBTPS2*, and *INSIG1*). The only mutation found that could explain the disease and co-segregates with the disease in the family was c.-405 A > G *SREBF2*. This genetic variation is located in the promoter region, where it affects transcription factor binding and increases *SREBF2* expression in different cell lines and at different levels. The SREBP system has been implicated in glucose and cholesterol metabolism, but it is mainly involved in cholesterol metabolism [10]. Recent works have shown the importance of *SREBF2* with respect to glucose metabolism [11]. Only few previous studies have found an association between the *SREBF2* gene and metabolic alterations [12,13,14,15]. The findings of this work indicated that a moderate increase in *SREBF2* gene expression may be involved in increasing blood levels of LDL cholesterol, total cholesterol, and glucose, which depends on the increased activation of different cell types.

The last work, written by Wu et al. [16], showed the importance of Calcium Channel Subunit Gamma-4 (*CaVγ4* or *CACNG4*) in glucose-stimulated insulin secretion. The work demonstrated the involvement of *CaVγ4* in blood glucose levels and its relation to age and gender. Based on the concept of PBC (pancreatic beta cell) de-differentiation precipitated by the loss of *CaVγ4* activity via MafA [17], this study showed that *CaVγ4* loss is related to altered insulin secretion due to loss of PBC identity, which is mediated by the suppression of CaMKII expression. These results support the importance of the *CaVγ4* gene in glucose metabolism and in PBC function. Therefore, this gene may be involved in glucose metabolism alterations, including in diabetes, and may be a target for diabetes treatment.

In conclusion, this Special Issue has allowed us to showcase different studies related to different metabolic alterations and the mechanisms involved, offering interesting results in this field. The importance of this research is growing due to the greater incidence of these diseases around the world and the complexity and variety of the mechanisms involved.

## Data Availability

No new data were created or analysed in this study. Data sharing is not applicable to this article.
